# Refraction and Motility of the Eye

**Published:** 1910-11

**Authors:** 


**Refraction and Motility of the Eye.** By **Ellice M. Alger**, M.D., Adjunct Professor of Ophthalmology at the New York Postgraduate Medical School. Octavo, pp. 122. With illustrations. Philadelphia: F. A. Davis Co. 1910. (Cloth, $2.00.)

This manual will take rank among the best on the subject of which it treats. A large experience has enabled the author to write interestingly and authoritatively along lines very much neglected by the physician in general practice. In the introduction the statement is made that whether the man who is not a specialist in eye work desires to enter the special field or not, he will be compelled to learn something of refraction in order to meet the requirements of his own patients and to hold them as against advertising irregulars of all sorts, who have taken up the business of fitting glasses. This little book is a good one to buy and to study. J. A. R.

